# A Prognostic Model of Pancreatic Cancer Based on Ferroptosis-Related Genes to Determine Its Immune Landscape and Underlying Mechanisms

**DOI:** 10.3389/fcell.2021.746696

**Published:** 2021-11-08

**Authors:** Xiao Yu, Qingyuan Zheng, Menggang Zhang, Qiyao Zhang, Shuijun Zhang, Yuting He, Wenzhi Guo

**Affiliations:** ^1^ Department of Hepatobiliary and Pancreatic Surgery, The First Affiliated Hospital of Zhengzhou University, Zhengzhou, China; ^2^ Key Laboratory of Hepatobiliary and Pancreatic Surgery and Digestive Organ Transplantation of Henan Province, The First Affiliated Hospital of Zhengzhou University, Zhengzhou, China; ^3^ Open and Key Laboratory of Hepatobiliary and Pancreatic Surgery and Digestive Organ Transplantation at Henan Universities, Zhengzhou, China; ^4^ Henan Key Laboratory of Digestive Organ Transplantation, Zhengzhou, China

**Keywords:** pancreatic cancer, ferroptosis, prognostic, immune, ECM

## Abstract

Pancreatic cancer is one of the malignant tumors with the worst prognosis in the world. As a new way of programmed cell death, ferroptosis has been proven to have potential in tumor therapy. In this study, we used the TCGA-PAAD cohort combined with the previously reported 60 ferroptosis-related genes to construct and validate the prognosis model and in-depth analysis of the differences in the function and immune characteristics of different RiskTypes. The results showed that the six-gene signature prognostic model that we constructed has good stability and effectiveness. Further analysis showed that the upregulated genes in the high-risk group were mainly enriched in extracellular matrix receptor-related pathways and other tumor-related pathways and the infiltration of immune cells, such as B, T, and NK cells, was suppressed. In short, our model shows good stability and effectiveness. Further studies have found that the prognostic differences between different RiskTypes may be due to the changes in the ECM-receptor pathway and activation of the immune system. Additionally, ICI drugs can treat pancreatic cancer in high-risk groups.

## Introduction

Pancreatic adenocarcinoma (PAAD) is one of the most aggressive and malignant tumors in humans. The prognosis of PAAD patients is inferior, with a median survival time of less than 6 months (Maisonneuve, 2019). Although pancreatic cancer is not common, owing to its high mortality rate, PAAD has become the seventh leading cause of cancer-related death worldwide, and the incidence of PAAD increases yearly (Rahib et al., 2014; Guarneri et al., 2019). Although surgical treatment, radiotherapy, and chemotherapy have made significant progress in decades, the prognosis of PAAD patients is still not optimistic because the molecular mechanism of this cancer has not been studied clearly (Jeune et al., 2019; Springfeld et al., 2019). Therefore, it is urgent to explore the pathogenic mechanism of PAAD from the molecular and genetic level and to find new therapeutic targets.

Ferroptosis is a new type of iron-dependent programmed cell death that is different from apoptosis, necrosis, and autophagy (Dixon et al., 2012). The primary mechanism of ferroptosis is that under the action of divalent iron or esteroxygenase, unsaturated fatty acids highly expressed on the cell membrane undergo liposomal peroxidation, thereby inducing cell death (Stockwell et al., 2017; Hassannia et al., 2019). Because of the unique role of ferroptosis in controlling programmed cell death, the role of ferroptosis in cancer and cancer treatment has been intensively investigated (Yuan et al., 2016; Liang et al., 2019). Studies have reported that the depletion of the intracellular iron storage prevents the oxidative stress induced by sorafenib in HCC cells, thus affecting the antitumor effect of sorafenib (Louandre et al., 2013; Louandre et al., 2015). Additionally, Sun *et al.* proved that heat shock protein β-1 (HSPB1) is a negative regulator of ferroptosis in cancer cells. Heat shock pretreatment and HSPB1 overexpression inhibited erastin-induced ferroptosis. In short, the unique role of ferroptosis in cancer is widely accepted by researchers (Friedmann Angeli et al., 2019). Dozens of genes related to ferroptosis have been identified (Louandre et al., 2015; Sun et al., 2016). However, the overall role of these genes in the progression of PAAD and their effect on prognosis are still unclear.

This study collected 60 ferroptosis-related genes (FRGs) previously reported in the literature and used the TCGA-PAAD cohort to construct a prognostic model of FRGs. A validation of internal and external datasets confirms the validity and stability of our model. Subsequently, various functional enrichment analyses were conducted to determine the underlying mechanism of the ferroptosis gene in PAAD. Additionally, we analyzed immune differences in models and explored the role of immunity in the differential prognosis caused by ferroptosis. Thus, we believe that this study plays a unique role in fully understanding the role of FRGs in PAAD and finding potential therapeutic targets.

## Materials and Methods

### Source of Expression Profile Data

The latest RNA-Seq data and clinical follow-up information were from the TCGA-PAAD cohort, and the download time was January 30, 2021. The GEO data were downloaded from Gene Expression Omnibus (GEO). GSE57495 and GSE71729 chip datasets with survival time were selected. The download time was January 30, 2021.

### Data Preprocessing

We processed the RNA-Seq data of TCGA-PAAD in the following steps:

1) Remove samples without clinical follow-up information, 2) remove samples without survival time, 3) remove samples without survival status, 4) convert Ensembl to gene symbol, and 5) take the median expression of genes with multiple gene symbols.

The following steps were processed for the GEO dataset:

1) Remove samples without clinical follow-up information, 2) remove samples without survival time and survival status, 3) convert the probe to gene symbol, 4) if one probe corresponds to multiple genes, remove the probe needle, and 5) take the median expression of genes with multiple gene symbols.

After preprocessing the three sets of data, we obtained 176 samples in TCGA-PAAD, 123 samples in the GSE71729 dataset, and 63 samples in GSE57495.

### Construction of a Prognostic Risk Model Based on Ferroptosis-Related Gene

We divide the 176 samples in TCGA-PAAD into a training set and validation set. To avoid the bias of random allocation affecting the stability of subsequent modeling, we prerandomize all samples 100 times without replacement and proceed according to the ratio of the training set: validation set = 1:1. The most suitable training set and validation set were selected according to the following conditions: 1) The two groups were similar in age distribution, gender, follow-up time, and the proportion of patient deaths; 2) after clustering the gene expression profiles of the two randomly grouped datasets. The number of samples in the two categories is close. Finally, we determined the best training set (n = 88) and validation set (n = 88). The sample information of the training set and the validation set was tested using the chi-square test (Table 1). The results showed that our grouping was reasonable, and there was no significant difference between the groups (*p* > 0.05). Subsequently, the single-factor and LASSO analysis of the training set was conducted. On the basis of the risk score, we constructed a risk model.

**TABLE 1:** Differences in clinical characteristics between training set and validation set.

Clinical features		TCGA-PAAD train	TCGA-PAAD text	P	Clinical features		TCGA-PAAD train	TCGA-PAAD text	P
OS	0	39	45	0.4505	Stage	I	7	14	0.3832
	1	49	43			II	75	70	
T stage	T1	5	2	0.09		III	1	2	
	T2	7	17			IV	3	1	
	T3	73	67			X	2	1	
	T4	1	2		Grade	G1	14	16	0.5703
	TX	2	0			G2	46	48	
N stage	N0	23	26	0.812		G3	27	21	
	N1	62	60			G4	0	2	
	NX	3	2			GX	1	1	
M stage	M0	35	44	0.2791	Gender	Male	47	49	0.8797
	M1	3	1			Female	41	39	
	MX	50	43		Age	≤65	47	46	1
						>65	41	42	

### Functional Enrichment Analysis

Differentially expressed genes were determined on the basis of the limma package. KEGG pathway analysis and GO functional enrichment analysis were conducted using R software package WebGestaltR (v.0.4.2), and the Gene set enrichment analysis (GSEA) analysis was based on the R software package GSVA for a single sample. GSVA is a popular R package, which was extensively utilized in cancer-related studies (Liu et al., 2021a; Liu et al., 2021b). All steps are shown in Supplementary Figure S1.

## Results

### Identification of Differentially Expressed FRGs With Prognostic Differences

We collected existing literature on ferroptosis and obtained 60 FRGs (Supplementary file S1) (Stockwell et al., 2017; Bersuker et al., 2019; Doll et al., 2019; Hassannia et al., 2019). Subsequently, for each FRG, the training set and survival data were used to construct a univariate Cox proportional hazard regression model using the R package survival coxph function, and *p* < 0.05 was considered a significant difference. As a result, seven differentially expressed FRGs with prognostic significance were identified: CD44, FANCD2, MT1G, PTGS2, SAT1, TFRC, and STEAP3.

### Regression Analysis of Least Absolute Shrinkage and Selection Operator

**FIGURE 1:**
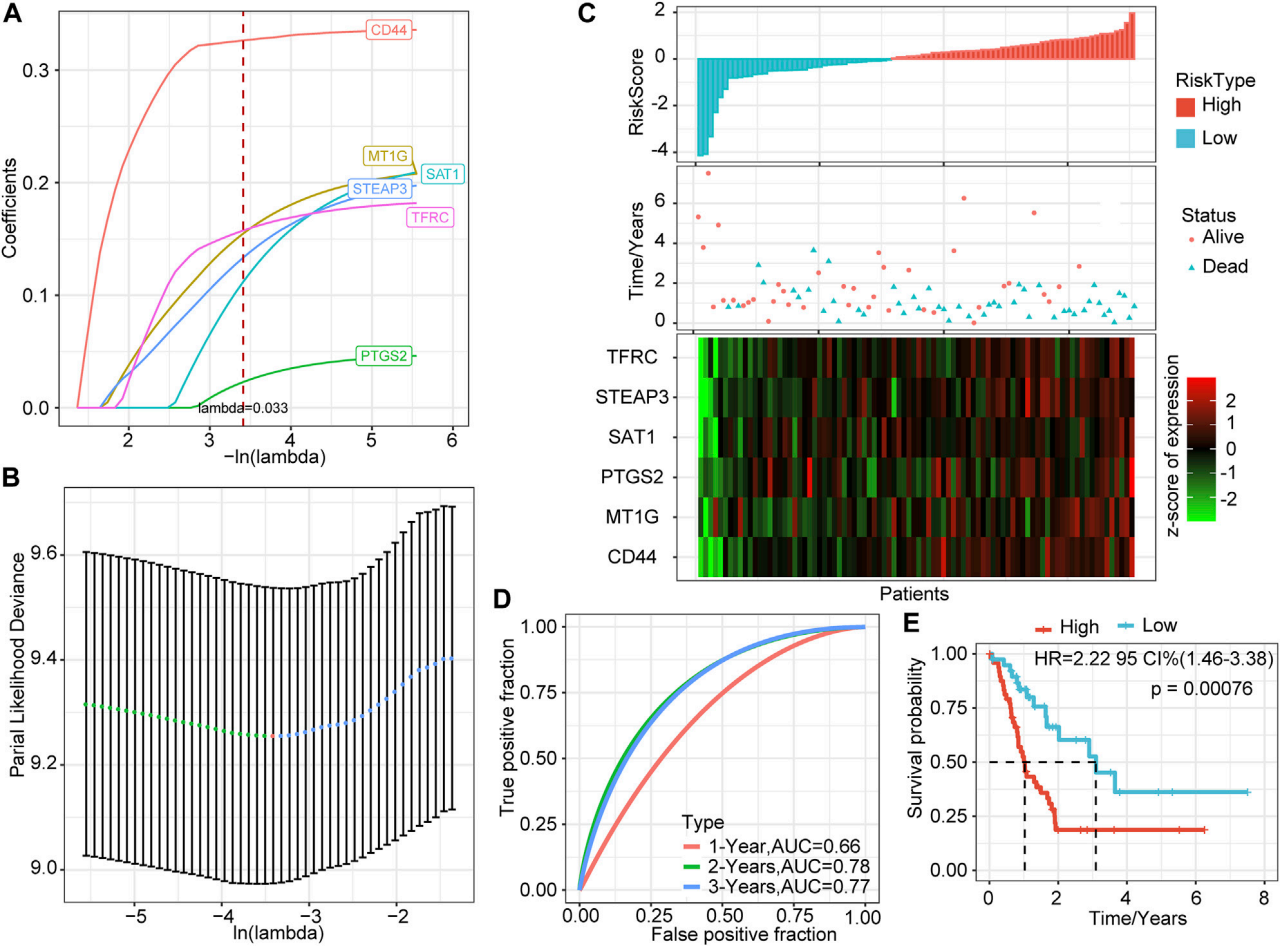
LASSO based on ferroptosis-related genes and prediction effect in the training set. **(A)** The changing trajectory of each independent variable; the horizontal axis represents the log value of the independent variable lambda, and the vertical axis represents the coefficient of the independent variable. **(B)** The confidence interval of each lambda. **(C)** RiskScore, survival time and status, and six-gene expression trend in the training set. **(D)** ROC curve of the prognostic model. **(E)** KM survival curve of the six-gene signature model in the training set.

The above seven genes were identified as related to the prognosis of PAAD patients. To further screen for key genes associated with the development and prognosis of PAAD, LASSO regression analysis was used to screen the above seven FRGs using the R software package “glmnet.” The trajectory of the coefficient of each gene with a value of −ln (lambda) is shown in Figure 1A. With the gradual increase in the lambda value, the number of coefficients of FRGs tending to 0 also gradually increased. We built the model by fivefold cross-validation, and the confidence interval under each lambda is shown in Figure 1B. The model was optimal when lambda = 0.033. Thus, we chose six genes when lambda = 0.033 as the model’s gene signature. Multifactor COX analysis on six genes was performed, and it calculated the risk coefficient of each gene and obtained the risk score calculation formula as follows:

RiskScore=0.340×CD44+0.216×MT1G+0.050×PTGS2+0.225×SAT1+0.186×TFRC+0.207×STEAP3

### Construction of a Prognostic Model Based on LASSO

We calculated the risk score of each sample based on the expression levels of the six genes identified by LASSO and plotted the distribution of risk scores (Figure 1C). Most samples in the training set had high-risk scores. The distribution of the survival status also showed that higher risk scores were associated with more death events. Interestingly, as the risk score increased, the expression levels of these six genes had a significant upward trend. Combined with the above formula, these results verified the tumor-promoting effect of FRGs in PAAD and the effectiveness of the six genes that we screened.

Further, we used the R software package timeROC to perform ROC analysis of prognostic predictions on the risk scores of the training set. The classification efficiency of prognostic predictions of 1, 2, and 3 years was analyzed (Figure 1D). The prediction performance of the classification model reached 0.66 (1 year), 0.78 (2 years), and 0.77 (3 years), which shows that our model had good classification performance.

To verify further the effectiveness of our model, we performed Z-score on risk score, divided the training set samples into high-risk groups (risk score >0) and low-risk groups (risk score <0), and showed the survival curve between the groups (Figure 1E). The results showed that the high-risk group had a significantly lower survival probability (*p =* 0.00076).

### The Validation Set in TCGA Verifies the Robustness of the Prognostic Model

**FIGURE 2:**
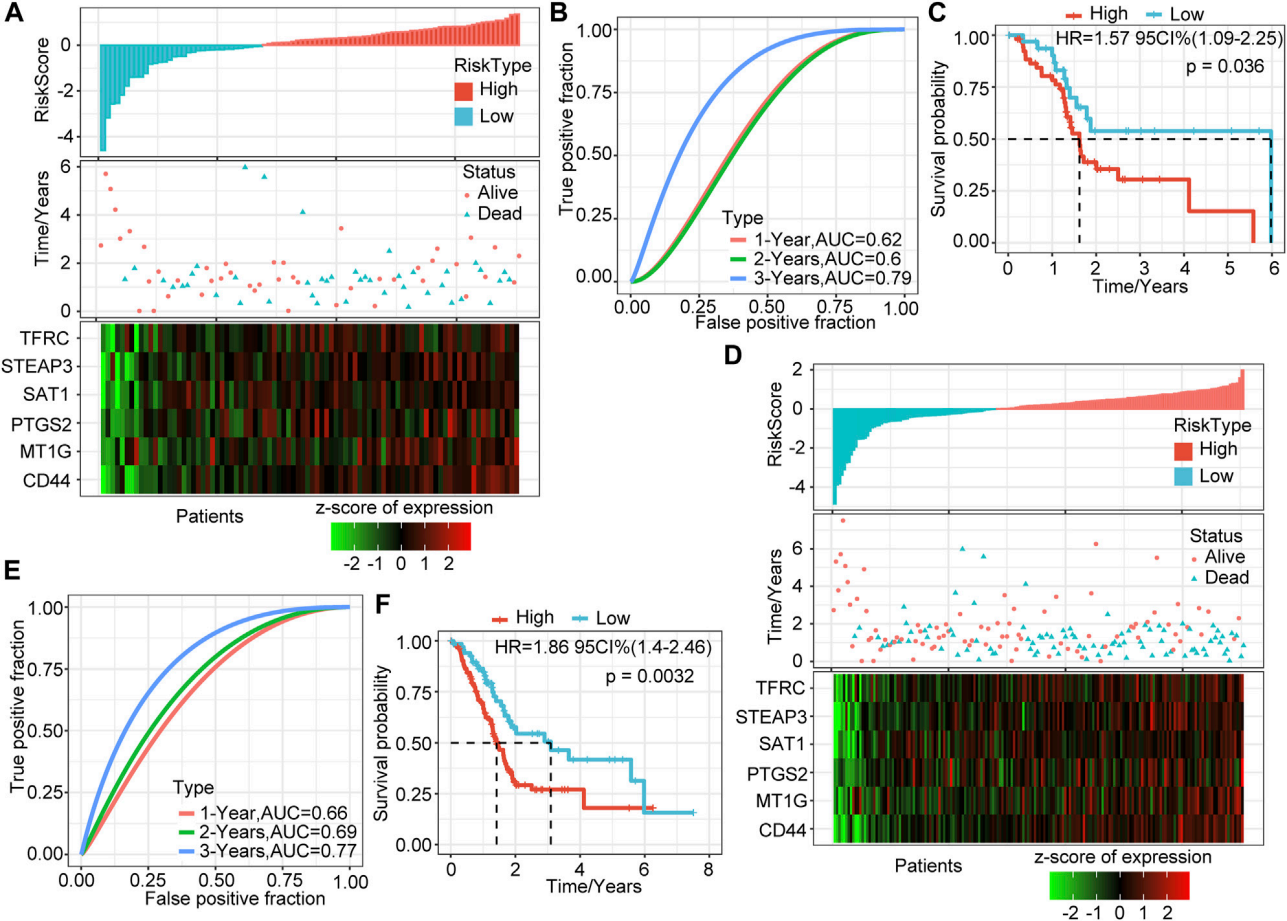
Validation of the prognostic model of the internal dataset. **(A)** RiskScore, survival time and status, and six-gene expression trend in the validation set. **(B)** ROC curve of the prognostic model in the validation set. **(C)** KM survival curve of the six-gene signature model in the validation set. **(D)** RiskScore, survival time and status, and six-gene expression trend in all sample sets (TCGA, 176 samples). **(E)** ROC curve of the prognosis model in all samples. **(F)** KM survival curve of the six-gene signature model in all samples.

To verify the robustness of the six-gene signature model, we calculated the risk score of each sample in the TCGA verification set based on the same model and coefficients as the training set and plotted the RiskScore distribution. Similar to the training set, higher risk scores correspond to more death events. The expression trends of these six genes were consistent with the training set (Figure 2A). ROC analysis showed that the model’s 1-, 2-, and 3-years AUCs in the validation set were 0.62, 0.6, and 0.79, respectively (Figure 2B). Finally, the prognosis of the high-risk group was significantly worse than that of the low-risk group (*p =* 0.036, Figure 2C).

We verified the above results in all samples of the TCGA-PAAD cohort. As we expected, as the risk score increased, the deaths of patients increased, and the expression levels of the six signature genes increased consistently (Figure 2D). The 1-, 2-, and 3-years AUCs of this model in all samples were 0.66, 0.69, and 0.77, respectively, showing an excellent long-term survival rate prediction (Figure 2E). The prognosis of the high-risk group was significantly worse than that of the low-risk group (*p =* 0.0032, Figure 2F). 106 samples were classified as high-risk groups, and 70 samples were classified as low-risk groups.

### External Dataset Verifies the Robustness of the Six-Gene Signature Model

**FIGURE 3:**
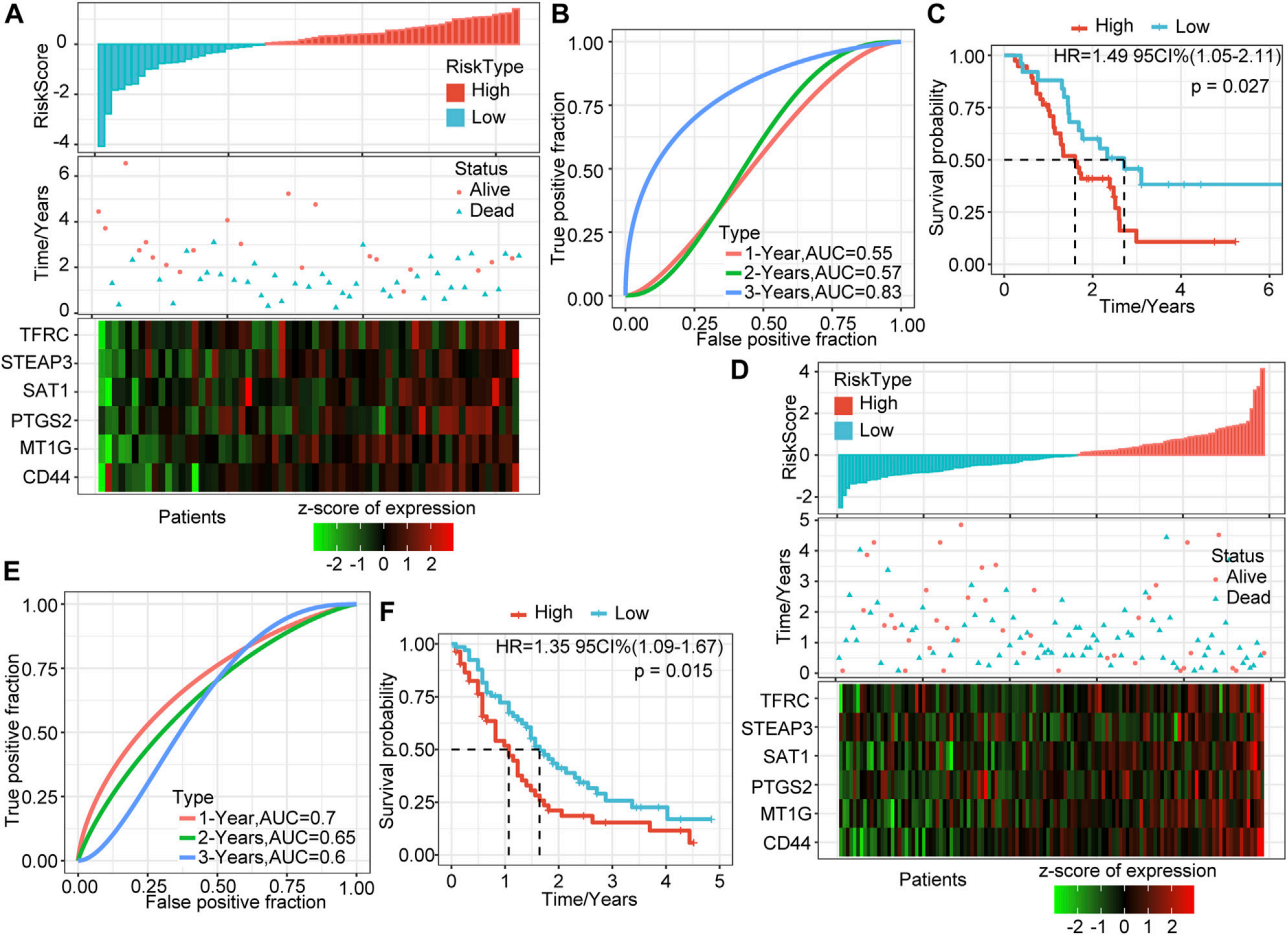
Validation of the prognostic model of the external dataset. **(A)** RiskScore, survival time and status, and six-gene expression trend in GSE57495. **(B)** ROC curve of prognosis model 1, 2, and 3 years. **(C)** KM survival curve of the six-gene signature model in GSE57495. **(D)** RiskScore, survival time and status, and six-gene expression trend in GSE71729. **(E)** ROC curve of prognosis model 1, 2, and 3 years. **(F)** KM survival curve of the six-gene signature model in GSE71729.

To determine further the validity and stability of our model, we conducted model verification on the GSE57495 and GSE71729 datasets. All parameters and tools were consistent with those in the training set. The RiskScore distribution of the independent verification dataset GSE57495 is shown in Figure 3A. Like the TCGA-PAAD cohort, most samples have high-risk scores, and these high-scoring samples have more death events and higher expression of the six signature genes. ROC analysis showed that the 1-, 2-, and 3-years AUCs of this model in GSE57495 were 0.55, 0.57, and 0.83, respectively, showing a good long-term survival prediction performance (Figure 3B). Survival analysis showed that consistent with the above results, there was a significant prognostic difference between the two groups (Figure 3C).

Subsequently, we conducted the above analysis in the GSE71729 dataset. The analysis results showed that the survival status of PAAD patients had an obvious relationship with the risk score, and the expression trends of the six signature genes have a strong consistency (Figure 3D). ROC analysis indicated that the 1-, 2-, and 3-years AUCs in the GSE71729 dataset were 0.7, 0.65, and 0.6, respectively (Figure 3E). Meanwhile, the survival analysis of the high-risk group and the low-risk group also showed significant differences. Like the performance in other datasets, the prognosis of the high-risk group was significantly worse (Figure 3F). However, most patients in this dataset had low-risk scores, perhaps due to the batch effect.

### Correlation Between Risk Score and Clinical Characteristics

**FIGURE 4:**
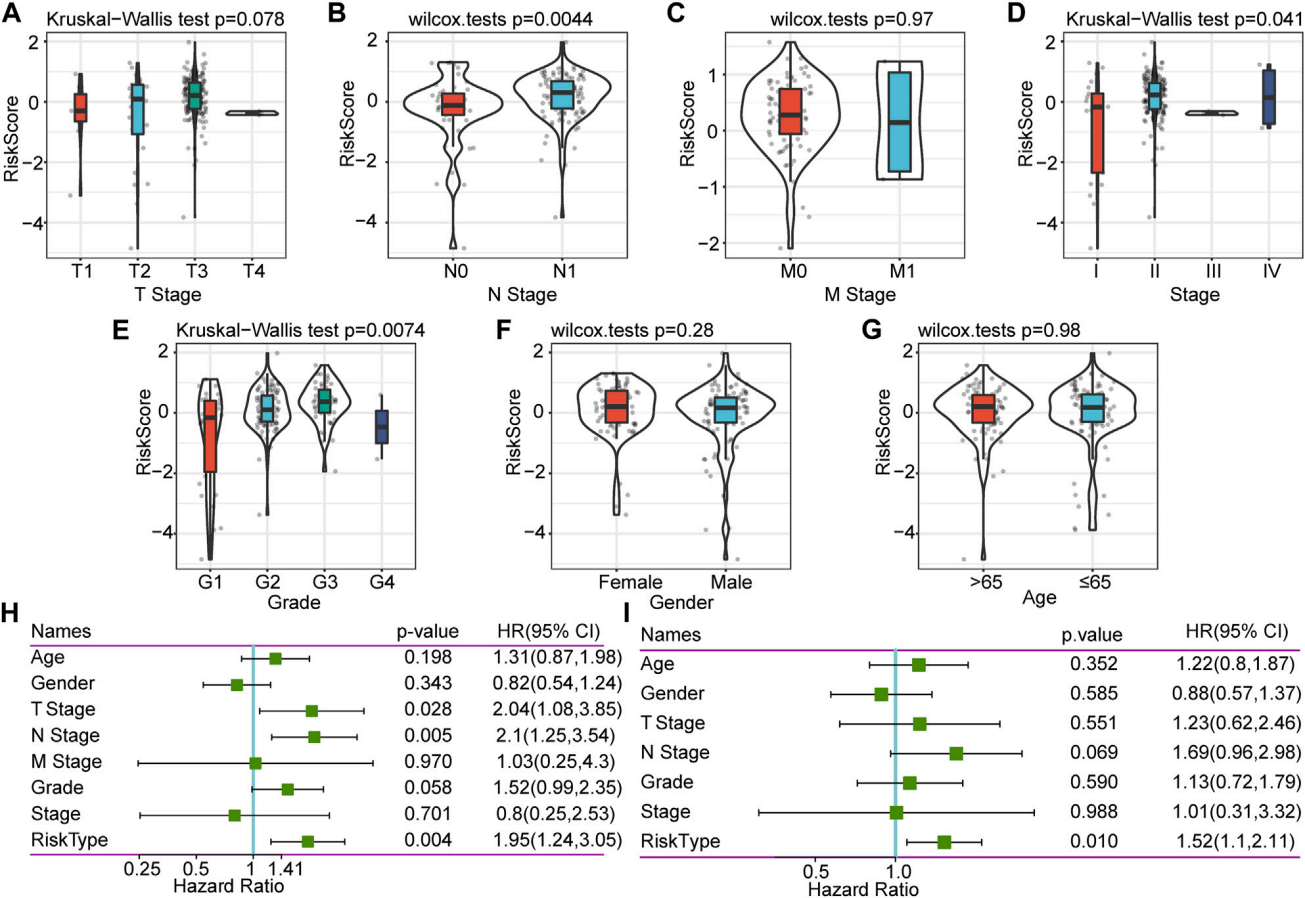
Correlation between RiskScore and clinical characteristics, and single-factor and multifactor analysis. **(A)** Correlation between RiskScore and T. stage. **(B)** Correlation between RiskScore and N. stage. **(C)** Correlation between RiskScore and M. stage. **(D)** Correlation between RiskScore and Stage. **(E)** Correlation between RiskScore and Grade. **(F)** Correlation between RiskScore and Gender. **(G)** Correlation between RiskScore and Age. **(H)** Clinical features and RiskScore’s single-factor analysis results. **(I)** Clinical features and RiskScore’s multivariate analysis results.

To explore further the characteristics of the risk score, we conducted an exploratory analysis of the risk score and clinical features. The results showed that there was no significant relationship between the risk score and T stage, M stage, gender, and age, and patients with different N stage, Stage, and grade have significantly different risk scores (Figures 4A–G). There was a clear trend here: higher risk scores were associated with a higher stage, and more differentiated samples have higher risk scores. Stage III and Grade 4 are inconsistent with other stages mainly because of the small sample size of these two stages, which results in large deviations.

### Single-Factor and Multivariate Analysis of Six-Gene Signature

To identify the independence of the six-gene signature model in clinical application, we performed single-factor and multifactor Cox regression analysis based on the clinical follow-up information of the TCGA database. These clinical indicators include age, gender, T stage, N stage, M stage, stage, grade, and our RiskType grouping information. Single-factor cox analysis results showed that T stage, N stage, and RiskType (*p =* 0.004, HR = 1.95) were significant risk factors for prognosis (Figure 4H). Multifactor Cox regression analysis showed that RiskType was an independent risk factor for prognosis (*p =* 0.01, HR = 1.52, Figure 4I). The above results indicate that our model has good predictive power in predicting the clinical prognosis of PAAD patients.

### Identification of Differentially Expressed Genes and Functional Enrichment Analysis

**FIGURE 5:**
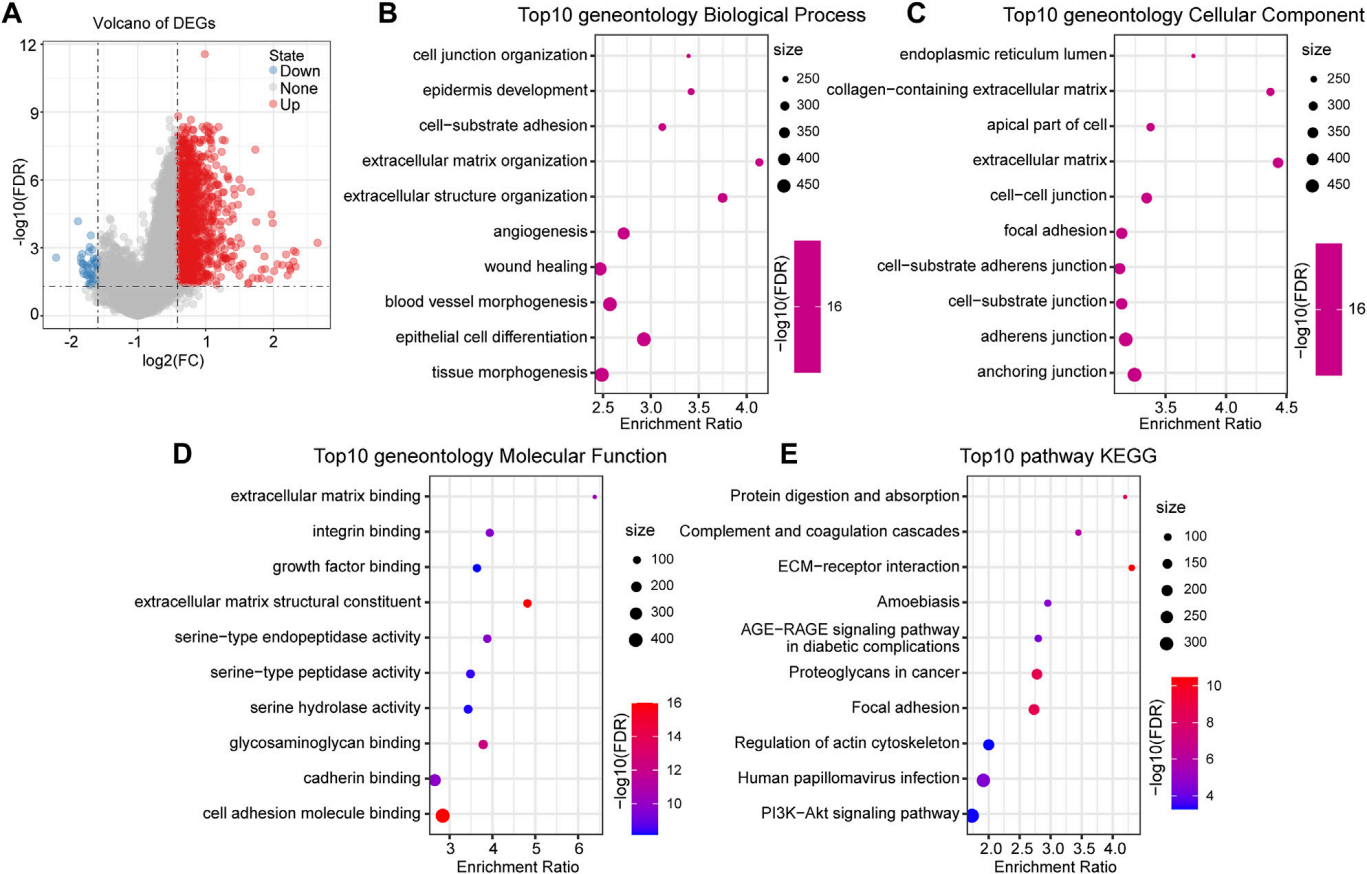
Functional enrichment analysis between RiskTypes. **(A)** Volcano plot of differentially expressed genes between high- and low-risk groups; the red dots represent upregulated genes in the high-risk group. **(B)** Top 10 BP terms of differentially upregulated genes between RiskTypes. **(C)** Top 10 CC terms of differentially upregulated genes between RiskTypes. **(D)** Top 10 MF terms of differentially upregulated genes between RiskTypes. **(E)** Top 10 KEGG pathways of differentially upregulated genes between RiskTypes.

We identified DEGs between the groups to understand the underlying mechanism of high- and low-risk groups with different prognoses. A total of 1,287 upregulated genes and 42 downregulated genes were identified in the high-risk group (Figure 5A). DEGs in the high-risk group were mainly upregulated expressed genes.

Subsequently, functional enrichment analysis on DEGs was performed using the R software package WebGestaltR (v0.4.2). With FDR <0.05 as the threshold, 1057 GO terms were annotated to biological processes (BP), 62 terms were annotated to molecular functions (MF), and 126 terms were annotated to cellular components (CC). The results showed that multiple pathways related to cell migration and tumor progression were enriched, including angiogenesis and epidermal development. Interestingly, pathways such as cell–cell and cell–substrate junctions were enriched in multiple categories. This may mean that the connection between tumor cells and cells or tissues is disturbed, which affects the tumor’s ability to migrate. Additionally, KEGG pathway enrichment analysis results showed that tumor-related pathways such as ECM-receptor interaction, focal adhesion, and PI3K-Akt signaling pathway were significant. We respectively showed the 10 most significantly enriched terms in each category (Figures 5B–E).

### GSEA of DEGs

**FIGURE 6:**
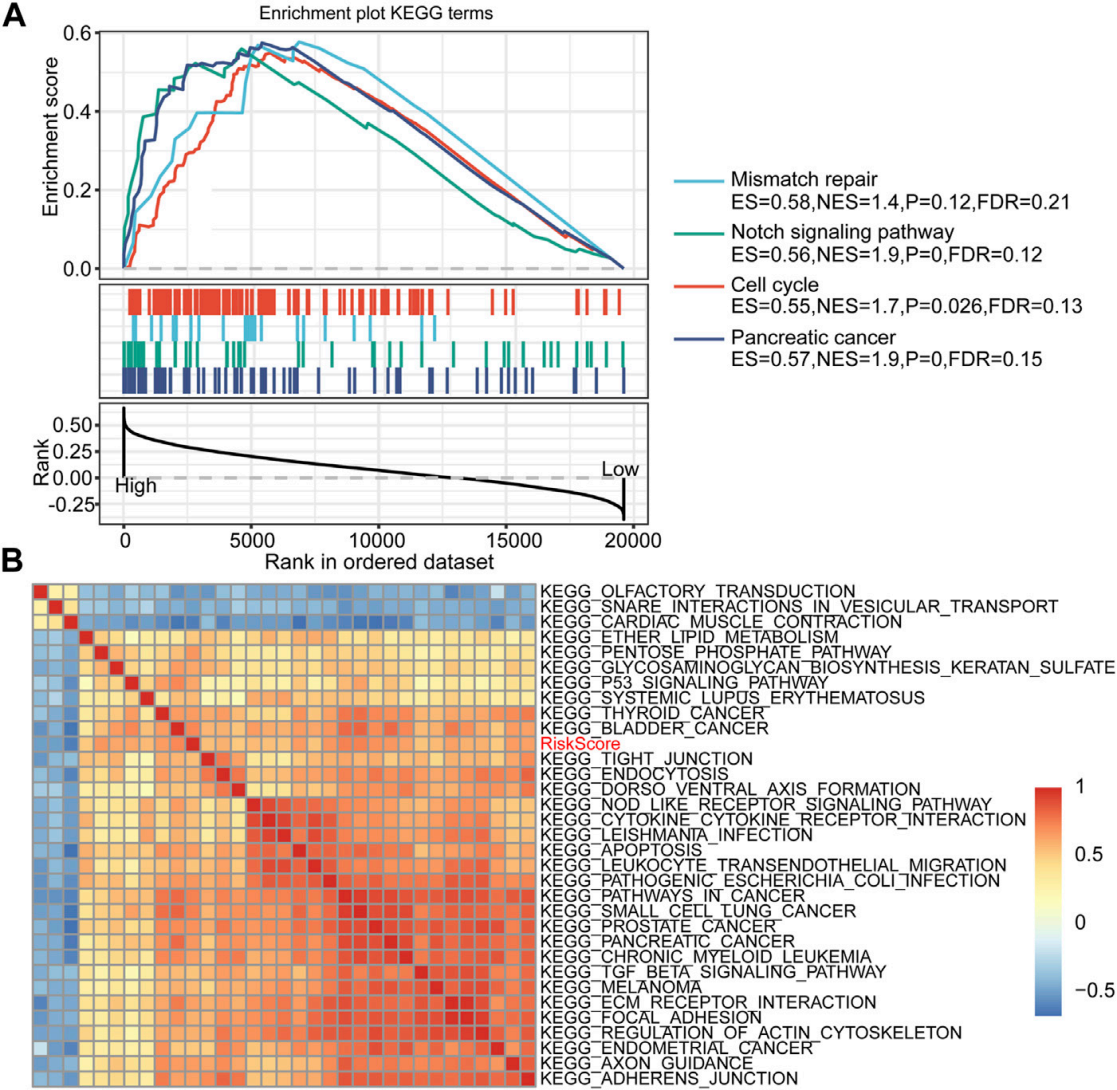
RiskScore-related pathways and GSEA results. **(A)** GSEA results between high-risk and low-risk groups. **(B)** Clustering correlation coefficients between the KEGG pathway and RiskScore with correlation >0.5.

We performed GSEA on the high-risk and low-risk groups, and the thresholds for the enrichment pathway selection were *p* < 0.05 and FDR <0.25 (Figure 6A). As we expected, multiple tumor-related pathways were enriched in the high-risk group, such as MISMATCH_REPAIR, NOTCH_SIGNALING_PATHWAY, CELL_CYCLE, and PANCREATIC_CANCER, which may imply that the poor prognosis of the high-risk group was a combination of multiple tumor pathways.

### Gene Expression Difference in Tumor-Related Pathways Between Groups

Furthermore, we performed a single-sample GSEA on the TCGA-PAAD cohort samples and calculated the ssGSEA score of each sample on different pathways. Correlation analysis of ssGSEA and risk scores was performed, and pathways with a correlation coefficient >0.5 were displayed (Figure 6B). A total of 32 pathways were screened, of which the ssGSEA of 29 pathways was positively correlated with the risk score, and the remaining three pathways were negatively correlated with the risk score. After consulting related literature, we found that multiple tumor-related pathways, including KEGG_PROSTATE_CANCER, KEGG_ECM_RECEPTOR_INTERACTION, and KEGG_FOCAL_ADHESIO, increased with the increase of RiskScore score whereas KEGG_SNARE_INTERACTIONS_IN_VESICULAR_TRANSPORT, KEGG_OLFACTORY_TRANSDUCTION, and KEGG_CARDIAC_MUSCLE_CONTRACTION had an opposite trend. Interestingly, the ECM-receptor pathway has been identified in multiple functional enrichment analyses. The expression of related genes in this pathway tended to increase as the risk score increased. Thus, the ECM-receptor pathway may be potentially linked to FRGs.

### Differences in Immune Infiltration Between Groups

**FIGURE 7:**
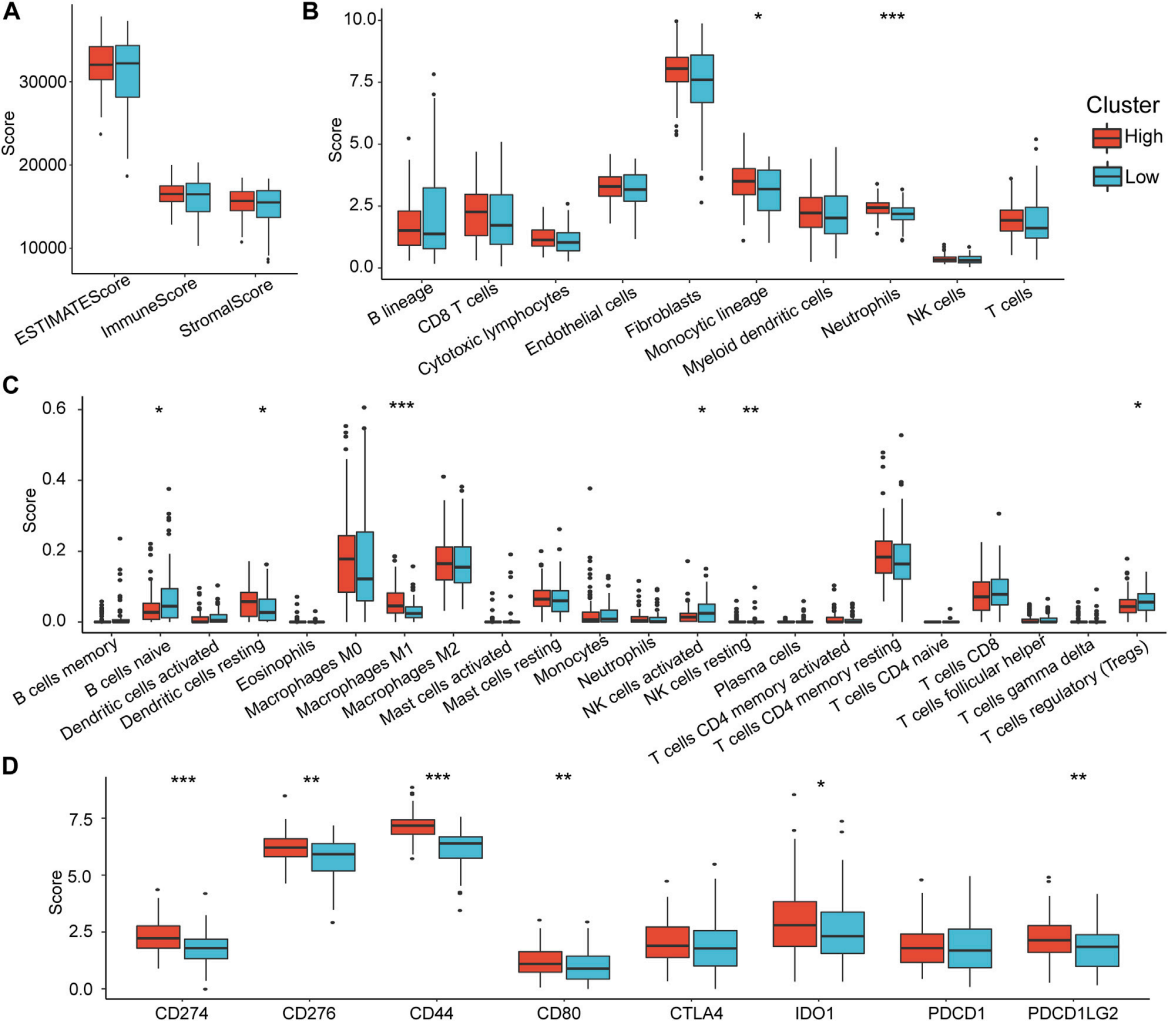
Differences in immune infiltration characteristics between RiskTypes. **(A)** Differences in Stromal Score, Immune Score, and ESTIMATE Score among RiskTypes. **(B)** Differences in 10 immune cells assessed by MCPcounter among RiskTypes. **(C)** The difference of 22 immune cell scores between RiskTypes using COBERSORT. **(D)** Differences in immune checkpoint genes between high- and low-risk groups.

To explore the differences in immune infiltration between the high-risk and low-risk groups that we identified, we assessed the differences in overall immune infiltration and immune cells using ESTIMATE, MCPcounter, and CIBERSORT tools. The results showed no significant differences in Stromal Score, Immune Score, and ESTIMATE Score between groups (Figure 7A). However, in the MCP method, monotypic lineage and neutrophils were significantly increased in the high-risk group (*p* < 0.05), which implies that the high-risk group has a stronger inflammatory response (Figure 7B). In the results of CIBERSORT, native B cells, activated NK cells, and Tregs infiltrate in the high-risk group were lower than those in the low-risk group, which implies that compared to low-risk group, the specific and nonspecific immune responses of the high-risk group were suppressed (Figure 7C).

Subsequently, we compared the expression differences in some immune checkpoints in the high- and low-risk groups. As we expected, almost all immune checkpoint genes were upregulated in the high-risk group. CD274, CD276, CD44, CD80, IDO1, and PDCD1LG2 had significant statistical differences (Figure 7D). This indicates that immune checkpoint-related pathways play an essential role in the poor prognosis of the high-risk group, suggesting that immune checkpoint inhibitors (ICIs) are effective for this type of pancreatic cancer.

### Comparison of Risk Models and Existing Models

**FIGURE 8:**
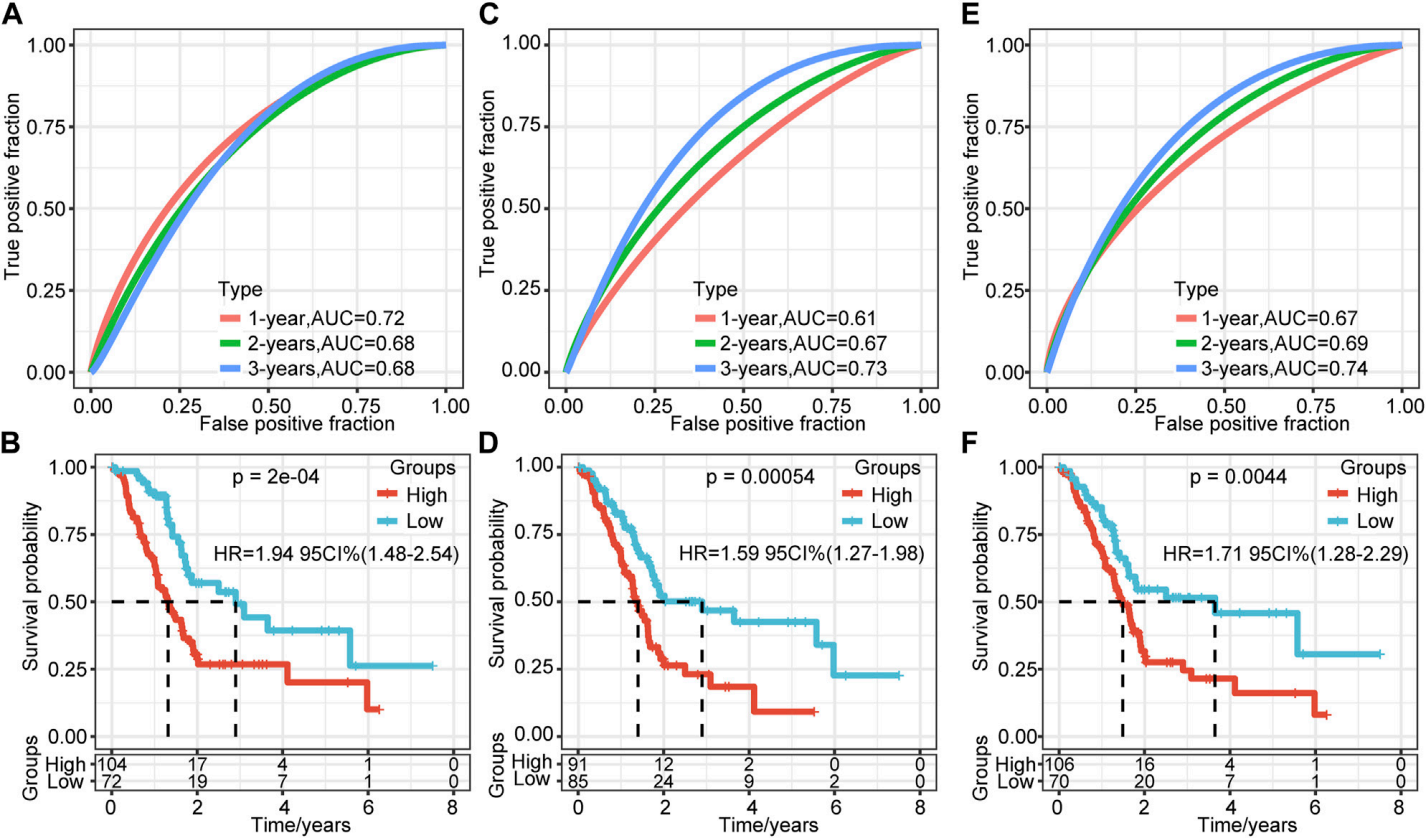
Comparison of the risk model and existing model. **(A–B)** ROC of seven-gene signature (Cheng) risk model and KM curve of high/low RiskTypes (Cheng). **(C–D)** ROC of the six-gene signature (Stratford) risk model and KM curve of high/low RiskTypes. **(E–F)** ROC of the nine-gene signature (Xu) risk model and KM curve of high/low RiskTypes.

To verify further the effectiveness of our model, by consulting relevant literature, we compared the predictive performance of three prognostic-related risk models (seven-gene signature (Cheng), six-gene signature (Stratford), and nine-gene signature (Xu)) and our model. To make the models comparable, we calculated the Z-score risk score of each PAAD sample based on the signature genes in these three models using the same method and divided the samples into the high-risk (risk score >0) and low-risk (risk score <0) groups. The ROC results of the seven-gene signature (Cheng) risk model showed that the 1-, 2-, and 3-years AUCs of the model were 0.72, 0.68, and 0.68, respectively (Figure 8A) (Cheng et al., 2019). The AUCs of the six-gene signature (Stratford) risk model were 0.61, 0.67, and 0.73, respectively (Figure 8C), and the AUCs of the nine-gene signature (Xu) risk model were 0.67, 0.69, and 0.74, respectively (Figure 8E) (Stratford et al., 2010; Xu et al., 2021). The prognosis of the three models is significantly different between the groups (Figures 8B,D,F). We found that the 1-, 2-, and 3-years AUCs of these three models on the TCGA data were lower than those of our model, indicating that our model had a good predictive performance.

## Discussion

Owing to the unique cell death mechanism of ferroptosis and its potential therapeutic prospects in cancer, ferroptosis has attracted the attention of many researchers (Yang et al., 2014; Conrad et al., 2016). Although the execution of ferroptosis requires the oxidation of polyunsaturated fatty acids, the underlying mechanism of the sensitivity of carcinogenic mutations and other noncarcinogenic cancer-related states to ferroptosis remains largely unclear. The latest research suggests that the tumor suppressor genes p53 and BCRA may be associated with increased sensitivity to ferroptosis (Li et al., 2012; Jiang et al., 2015; Wang et al., 2016). Additionally, Liu *et al.* found that in clear cell renal cell carcinoma, the deletion of the von-Hippel-Lindau gene makes this type of tumor sensitive to ferroptosis caused by glutathione depletion (Yang et al., 2014; Miess et al., 2018). Additionally, reports have pointed out that ferrophilic cancer cells may release immunomodulatory signals, such as lipid mediators, to attract immune cells, such as macrophages, for effective phagocytosis (Elliott and Ravichandran, 2016; Kloditz and Fadeel, 2019; Liu et al., 2021c). Although we have made positive progress in the mechanism that drives ferroptosis, ferroptosis in tumors and its regulatory mechanism are still contradictory (Friedmann Angeli et al., 2019). It is necessary to identify further the difference between ferroptosis that inhibits tumor growth and ferroptosis that drives cancer progression.

In this study, we used the TCGA-PAAD cohort to perform univariate cox regression combined with the previously reported 60 FRGs and identified seven prognostic-related ferroptosis genes. Subsequently, the LASSO algorithm was used to reduce dimensionality and construct a six-gene signature prognostic model. We verified the effectiveness of the model in the training set, the validation set, and all samples. The model’s long-term prognosis predicted that AUC reached 0.79. Additionally, to verify the stability of the model on different sequencing platforms, we confirmed it in GSE57495 and GSE71729. The results showed that whether it is an internal dataset or an external dataset, the model showed convincing stability and effectiveness. Subsequently, we analyzed the correlation between different risk groups and clinical characteristics. There were significant differences in N stage, Stage, and Grade between the risk groups. Additionally, there was a trend that the high-risk group has a higher degree of differentiation. Both univariate and multivariate COX regression analyses showed that RiskType was significantly related to prognosis. To validate further the prognostic model, three pre-existing PAAD prognostic models were compared with our model. The 1-, 3-, and 5-years AUCs of these three models on the same dataset were lower than those of our model. This also verifies that our model has prognostic prediction ability.

Further functional enrichment analysis showed that the ECM-receptor pathway and the cell–cell and cell–matrix connection pathways were enriched by multiple categories. The extracellular matrix (ECM) is composed of a complex mixture of structural and functional macromolecules and plays a vital role in the formation of tissues and organs and the maintenance of the structure and function of cells and tissues (Mohan et al., 2020). Cells interact with ECM through ECM receptors to control cell migration, differentiation, and apoptosis (Eble and Niland, 2019; Mohan et al., 2020). A study by Brown *et al.* found that ECM detachment is an essential factor in triggering the ferroptosis of cancer cells (Buchheit et al., 2014; Brown et al., 2017; Dixon, 2017). The activation of Src mediated by α6β4 contributes to resistance to ferroptosis. In the absence of α6β4, cell ECM detachment is prone to ferroptosis. Our research results corroborate this conclusion. However, there is also evidence that ECM detachment can increase intracellular reactive oxygen species (ROS) and cause ROS-dependent cell death (Schafer et al., 2009). It is essential to determine the difference between apoptosis and ferroptosis, which may determine the outcome of the cell, which requires more rigorous experiments.

Additionally, ferroptosis regulates the antitumor response of the immune system. There is evidence that different types of ferritic cancer cells can release HMGB1, a damage-related molecule, in a ferroptosis-dependent manner, and can then obtain the characteristics of immune stimulation and act as an adjuvant (Yamazaki et al., 2014; Yu et al., 2015; Wen et al., 2019). This molecule can promote the activation of innate and adaptive immune systems by binding with pattern recognition receptors. This conclusion is consistent with our research results (Liu et al., 2021d). We found that various immune cells, including B cells, helper T cells, and NK cells, were upregulated in the low-risk group. This implied that compared to the high-risk group, samples from the low-risk group could activate the specific and non-specific immune systems through the above pathways, then stimulate the anti-tumor response of immune system. Interestingly, we found a significant difference in the expression of immune checkpoints between the high-risk group and the low-risk group. Almost all immune checkpoint genes were upregulated in the high-risk group. This may mean that pancreatic cancer in the high-risk group suppresses the immune response by “hijacking” the immune checkpoint pathway to obtain immune escape (Liu et al., 2021e). This suggests that ICIs is an effective treatment for this type of pancreatic cancer with a worse prognosis.

Although many studies have explored the mechanism of ferroptosis and the biological processes that it causes, it cannot be ignored that ferroptosis is a kind of programmed cell death induced by multifactorial stress. We should explain this phenomenon from multiple perspectives. In our pancreatic cancer research, FRGs are involved in various tumor-related pathways. The differential prognosis of our model is the result of multiple tumor-related pathways, including the ECM-receptor pathway and tumor immune regulation. These results lay the foundation for further exploration of the role and mechanism of ferroptosis in pancreatic cancer.

## Conclusion

We constructed a six-gene signature prognostic model based on FRGs. After extensive verification, this model has been proven to be stable and effective in predicting the prognosis of pancreatic cancer. Further research showed that the prognostic differences between different RiskTypes may be due to the changes in the ECM-receptor pathway and activation of the immune system. ICI drugs can treat pancreatic cancer in the high-risk group in our model.

## Data Availability

Publicly available datasets were analyzed in this study, these can be found in The Cancer Genome Atlas (https://portal.gdc.cancer. gov/) and Gene Expression Omnibus (GSE57495, and GSE71729).

## Supplementary Material

Supplementary Figure S1 Research flowchart of ferroptosis-related genes in the prognostic model of pancreatic cancer.
