# Supplementary figures and images for: A Prognostic Model of Pancreatic Cancer Based on Ferroptosis-Related Genes to Determine Its Immune Landscape and Underlying Mechanisms
Source: Front Cell Dev Biol. 2021 Nov 8;9:746696. doi: 10.3389/fcell.2021.746696 (PMC8606410; doi:10.3389/fcell.2021.746696)

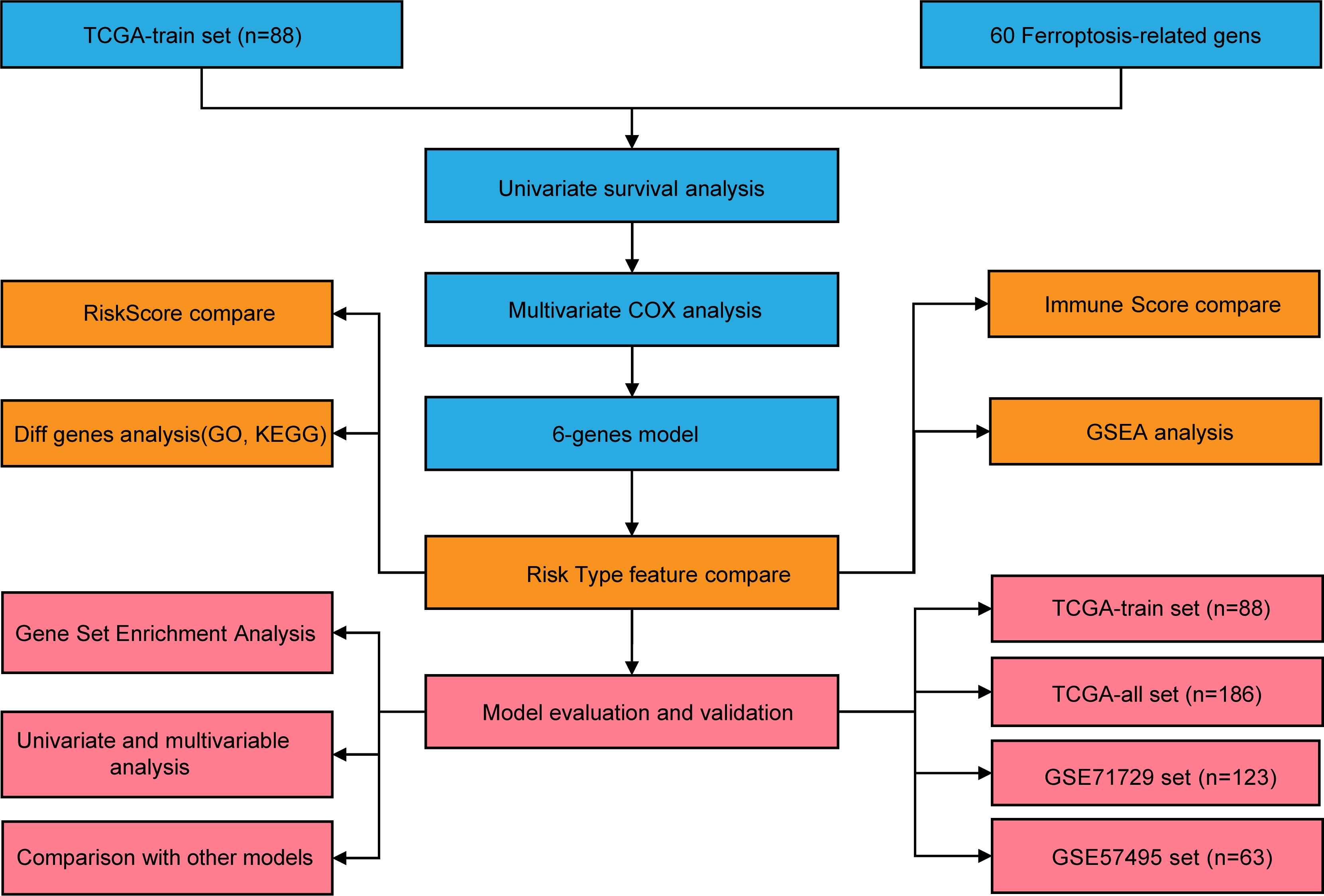

Supplement: Supplementary file 1 [file Image1.TIF]
